# Supplementary material for: Purgative Effect, Acute Toxicity, and Quantification of Phorbol-12-Myristate-13-Acetate and Crotonic Acid in Croton tiglium L. Seeds Before and After Treatment by Thai Traditional Detoxification Process
Source: Int J Mol Sci. 2025 Aug 9;26(16):7714. doi: 10.3390/ijms26167714 (PMC12386474; doi:10.3390/ijms26167714)
Supplement: Supplementary file 1 [file ijms-26-07714-s001.zip › ijms-3793939-supplementary.pdf]

Supplementary Materials

Purgative Effect, Acute Toxicity, and Quantification of Phorbol-12-Myristate-13-Acetate and Crotonic Acid in *Croton tiglium* L. Seed before and after Treatment by Thai Traditional Detoxification Process

Ronnachai Poowanna, Pawitra Pulbutr, Anake Kijjoa and Somsak Nualkaew

Figure S1. Structure of di-(ethylhexyl)phthalate.

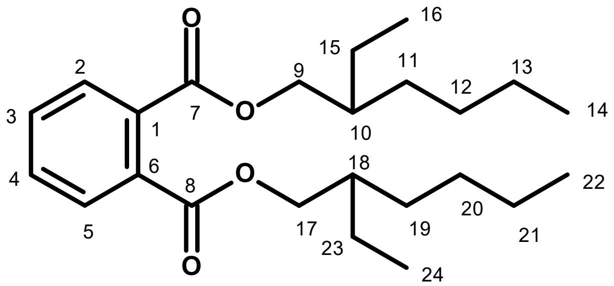

Figure S2. ^{1}H NMR data of di-(2-ethylhexyl)phthalate ($CDCl_3$, 400 MHz).

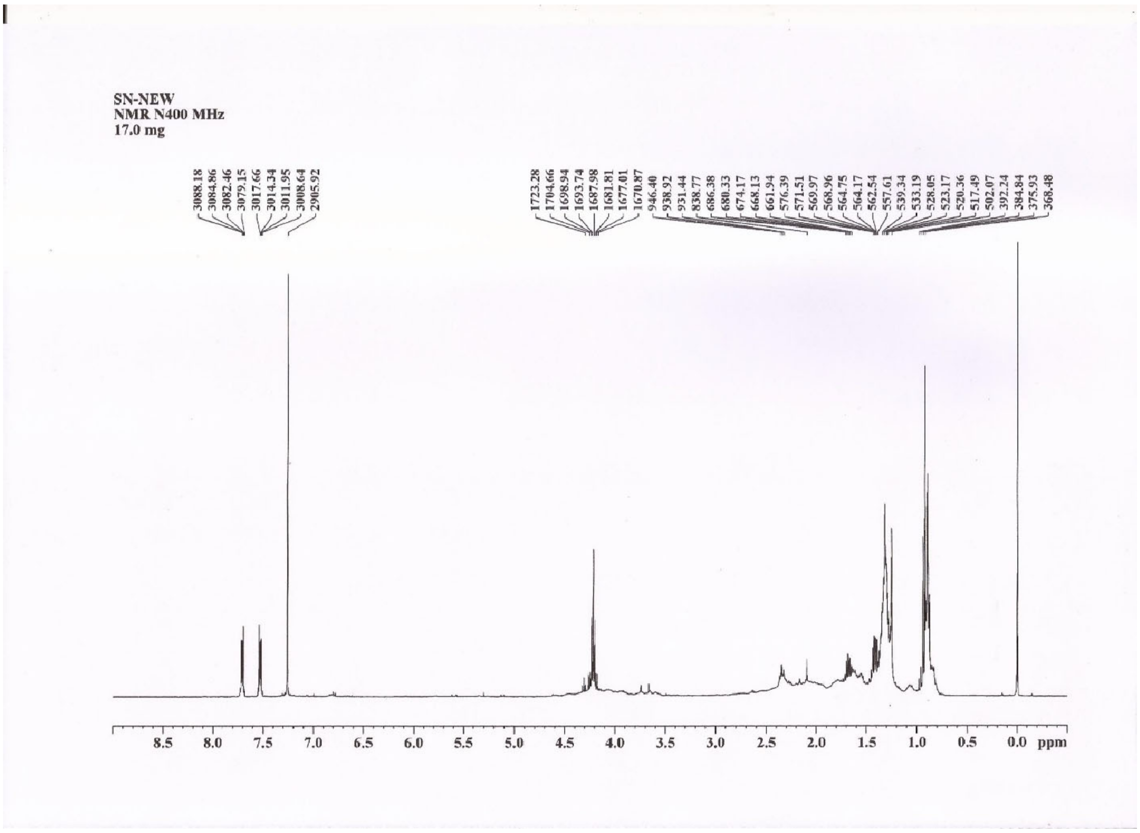

Figure S3: ^{13}C NMR data of di-(2-ethylhexyl)phthalate ($CDCl_3$, 100 MHz).

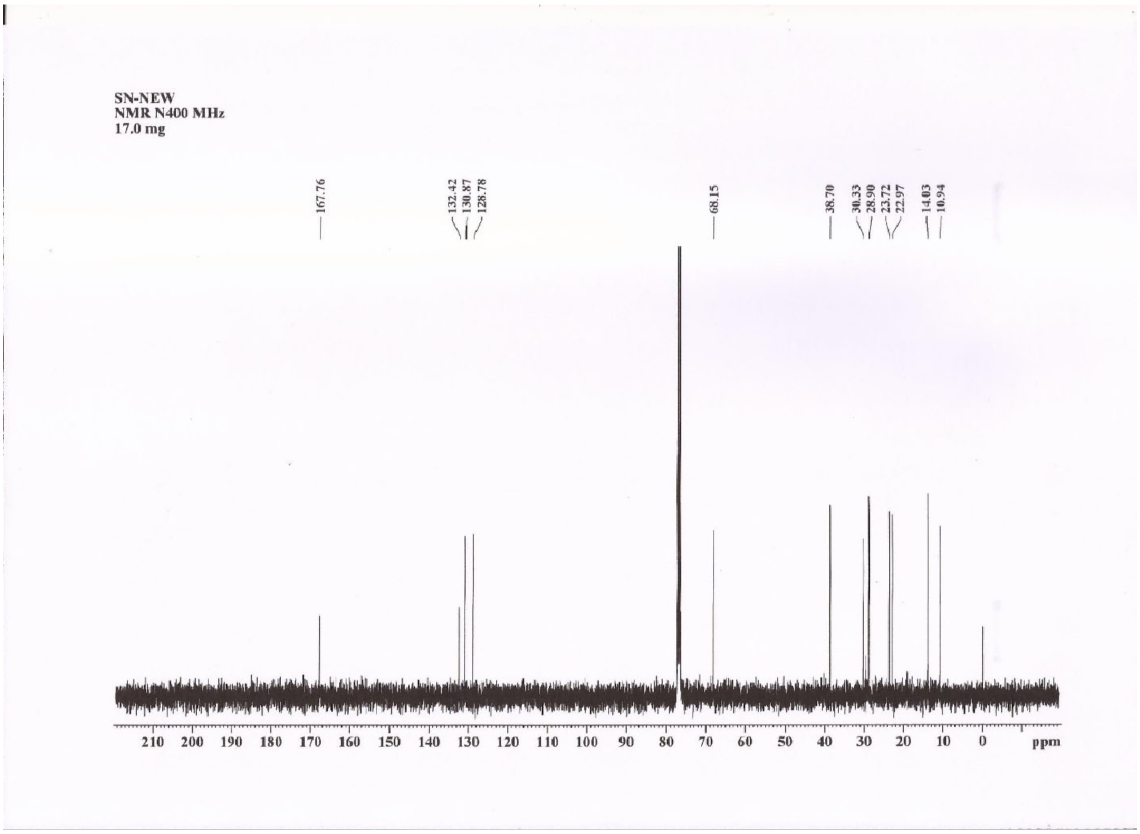

Figure S4. DEPT 135 spectrum of di-(2-ethylhexyl)phthalate ($CDCl_3$, 100 MHz).

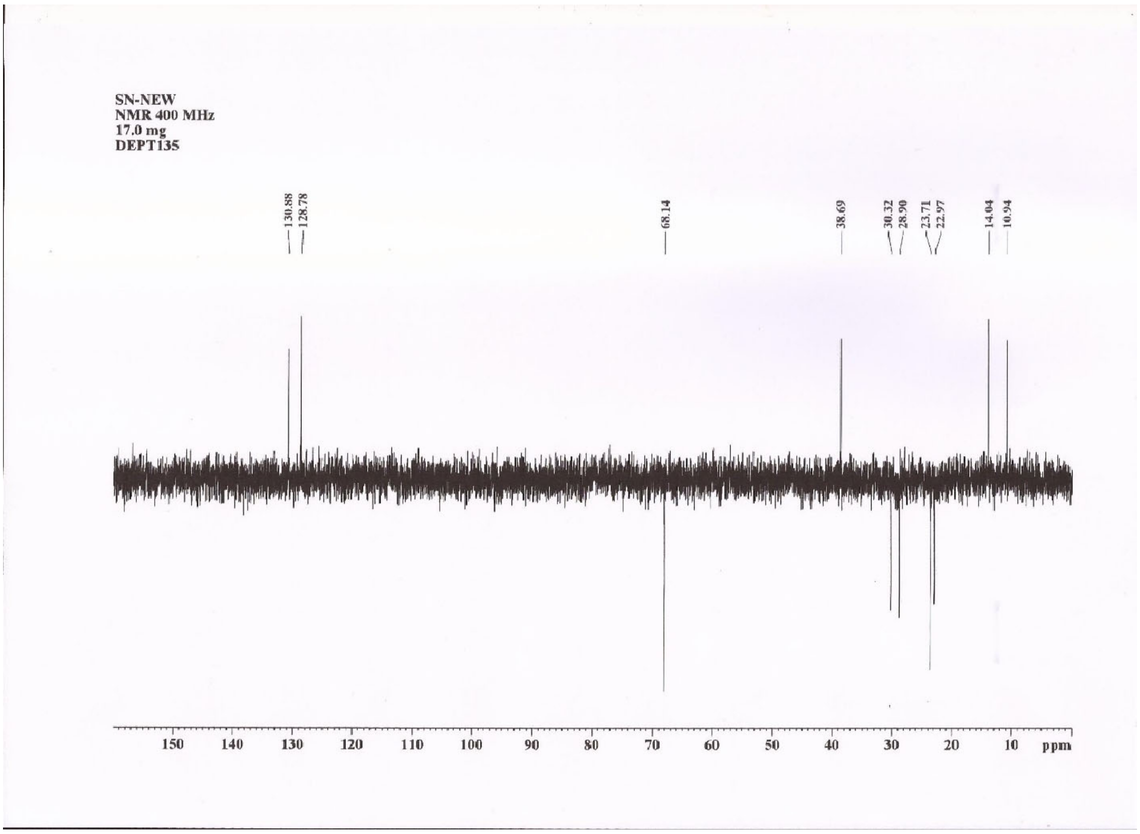

Figure S5. DEPT 90 spectrum of di-(2-ethylhexyl)phthalate ($CDCl_3$, 100 MHz).

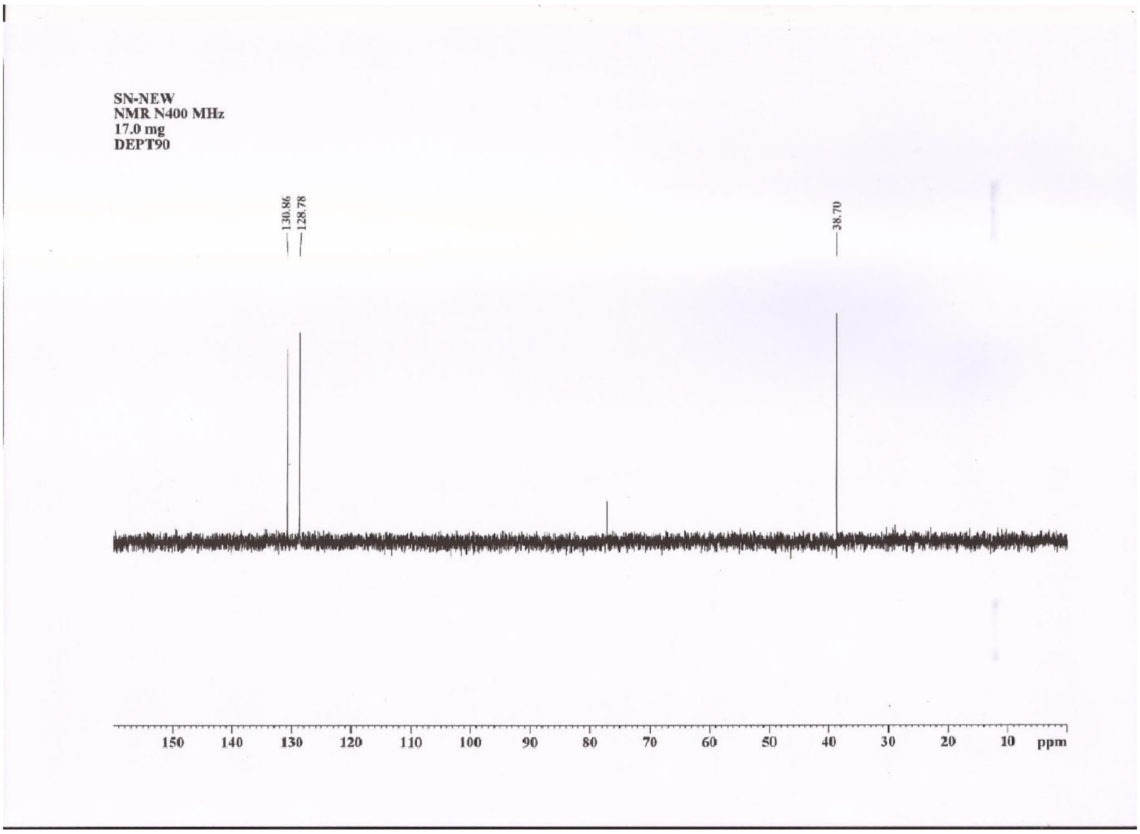

Figure S6. COSY spectrum of di-(2-ethylhexyl)phthalate ($CDCl_3$, 400 MHz).

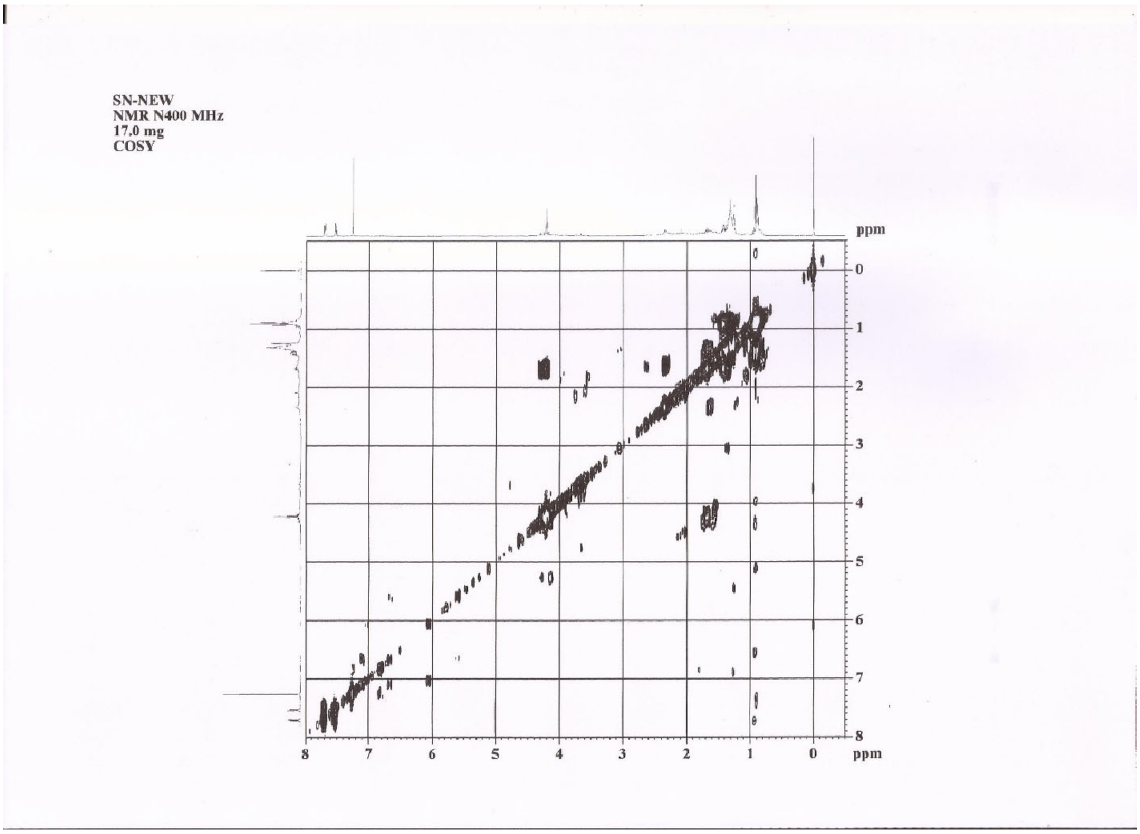

Figure S7. HSQC spectrum of di-(2-ethylhexyl)phthalate ($CDCl_3$, 400 MHz).

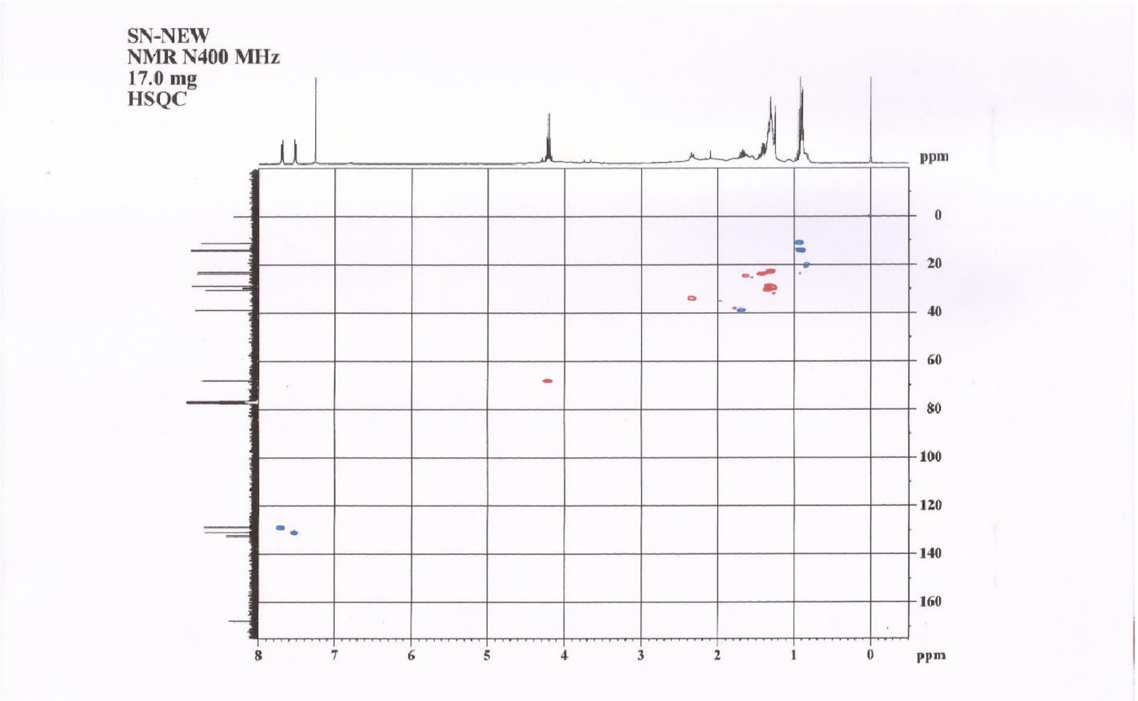

Figure S8. HMBC spectrum of di-(2-ethylhexyl)phthalate ($CDCl_3$, 400 MHz).

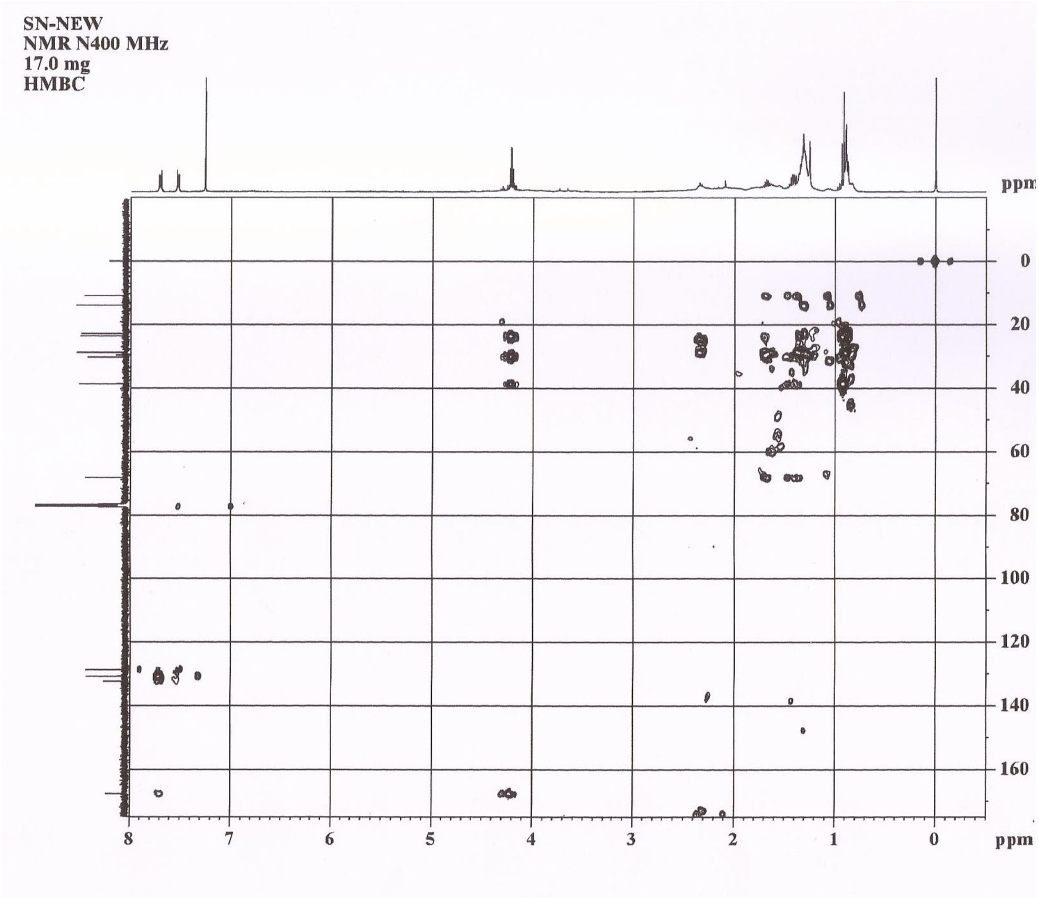

Figure S9. (+)-HRESIMS spectrum of di-(2-ethylhexyl)phthalate ($CDCl_3$, 400 MHz).

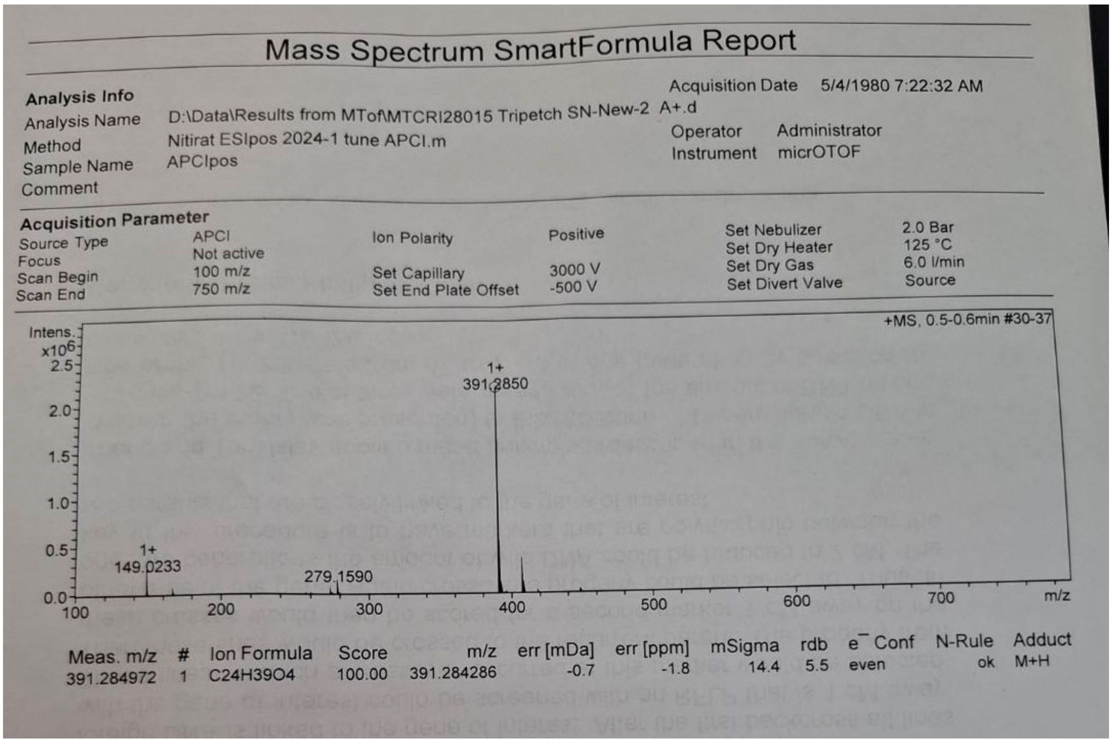

Table S1. ^{1}H and ^{13}C NMR data of di-(2-ethylhexyl)phthalate ($CDCl_3$, 400 and 100 MHz)

Position	δ_C, type	δ_H (*J* in Hz)*	COSY	HMBC
1 (6)	132.4, C	-		
2 (5)	128.8, CH	7.71, dd (5.8, 3.3)	H-3	
3 (4)	130.9, CH	7.53, dd (5.8, 3.3)	H-2	
7 (8)	167.8, CO	-		
9 (17)	68.2, CH_2	4.82, m	H-10	C-11, 15,
10 (18)	38.7, CH	1.70, m	H-9	C-9, 12
11 (19)	30.3, CH_2	1.35, m		C-12
12 (20)	28.9, CH_2	1.32, m		
13 (21)	23.0, CH_2	1.30, m		
14 (22)	14.0, CH_3	0.90, t (6.9)		C-12, 13,
15 (23)	23.7, CH_2	1.42, m		
16 (24)	10.9, CH_3	0.92 t (7.5)		C-C-10, 15

*assigned by HSQC
